# Characterization of a Genomic Region under Selection in Cultivated Carrot (*Daucus carota* subsp. *sativus*) Reveals a Candidate Domestication Gene

**DOI:** 10.3389/fpls.2017.00012

**Published:** 2017-01-18

**Authors:** Alicja Macko-Podgórni, Gabriela Machaj, Katarzyna Stelmach, Douglas Senalik, Ewa Grzebelus, Massimo Iorizzo, Philipp W. Simon, Dariusz Grzebelus

**Affiliations:** ^1^Institute of Plant Biology and Biotechnology, Faculty of Biotechnology and Horticulture, University of Agriculture in KrakowKrakow, Poland; ^2^Vegetable Crops Research Unit, United States Department of Agriculture-Agricultural Research Service, Department of Horticulture, University of Wisconsin–Madison, MadisonWI, USA; ^3^Plants for Human Health Institute, Department of Horticultural Science, North Carolina State University, KannapolisNC, USA

**Keywords:** AT-hook motif nuclear localized (AHL), domestication syndrome, genotyping-by-sequencing, linkage disequilibrium, single nucleotide polymorphism, storage root

## Abstract

Carrot is one of the most important vegetables worldwide, owing to its capability to develop fleshy, highly nutritious storage roots. It was domesticated ca. 1,100 years ago in Central Asia. No systematic knowledge about the molecular mechanisms involved in the domestication syndrome in carrot are available, however, the ability to form a storage root is undoubtedly the essential transition from the wild *Daucus carota* to the cultivated carrot. Here, we expand on the results of a previous study which identified a polymorphism showing a significant signature for selection upon domestication. We mapped the region under selection to the distal portion of the long arm of carrot chromosome 2, confirmed that it had been selected, as reflected in both the lower nucleotide diversity in the cultivated gene pool, as compared to the wild (π_w_/π_c_ = 7.4 vs. 1.06 for the whole genome), and the high F_ST_ (0.52 vs. 0.12 for the whole genome). We delimited the region to ca. 37 kb in length and identified a candidate domestication syndrome gene carrying three non-synonymous single nucleotide polymorphisms and one indel systematically differentiating the wild and the cultivated accessions. This gene, *DcAHLc1*, belongs to the AT-hook motif nuclear localized (AHL) family of plant regulatory genes which are involved in the regulation of organ development, including root tissue patterning. AHL genes work through direct interactions with other AHL family proteins and a range of other proteins that require intercellular protein movement. Based on QTL data on root thickening we speculate that *DcAHLc1* might be involved in the development of the carrot storage root, as the localization of the gene overlapped with one of the QTLs. According to haplotype information we propose that the ‘cultivated’ variant of *DcAHLc1* has been selected from wild Central Asian carrot populations upon domestication and it is highly predominant in the western cultivated carrot gene pool. However, some primitive eastern landraces and the derived B7262 purple inbred line still carry the ‘wild’ variant, reflecting a likely complexity of the genetic determination of the formation of carrot storage roots.

## Introduction

Carrot is one of the most important root vegetable crops grown worldwide on ca. 1.2 million hectares (FAOSTAT, 2013). Its progenitor, wild *Daucus carota* L., is a weed commonly occurring across continents in the temperate climatic zone. Asia Minor and the inner Asiatic regions have been indicated as likely centers of origin of the cultivated carrot (Vavilov, 1992). As a storage root similar to modern carrots, it has been grown in those regions since the 10th century (Mackevic, 1932; Zagorodskikh, 1939). The first domesticated carrots produced purple or yellow roots (Banga, 1963), while orange carrots did not appear in Europe before the 15th century (Banga, 1957a,b; Stolarczyk and Janick, 2011). Recent molecular studies have provided answers to questions concerning carrot evolution and domestication and confirmed that domesticated carrots were derived from wild populations of Central Asian *D. carota* (Iorizzo et al., 2013). It was also clearly shown that the cultivated germplasm could be divided into two distinct groups: the eastern and the western gene pools (Baranski et al., 2012; Iorizzo et al., 2013; Grzebelus et al., 2014), in agreement with an earlier hypothesis on carrot evolution by Small (1978), based on morphological observations.

Nevertheless, little information has been available on the molecular basis of domestication traits in carrot. To date, research on crop domestication has focused on staple food crops with little attention toward root vegetables (Meyer et al., 2012). Thus, traits such as loss of seed shattering, dormancy, and branching, referred to as the domestication syndrome, have been extensively studied (Zohary and Hopf, 2000). However, with respect to carrot and other root crops, the list of traits important for primary domestication should include, among others, the ability to form fleshy roots, minimal lateral root branching and biennial growth habit. Of these traits, only the *Vrn*1 locus responsible for early flowering, which was an apparent target for selection in the course of carrot domestication, has been recently mapped on carrot chromosome 2 (Alessandro et al., 2013). However, the gene has not been characterized. After primary traits have been selected and fixed, the process of domestication has often directed more attention to quality traits such as color, shape, flavor, and physiological traits contributing to uniformity (Doebley et al., 2006). Further improvement of carrot required selection for a range of traits determining root quality, e.g., shape, color, smoothness, etc. To date, only the genetic factors underlying carrot root color have been extensively investigated, leading to the identification and mapping of *Y* and *Y2* genes governing carotene accumulation (Bradeen and Simon, 1998) and *P1*, the gene involved in anthocyanin accumulation (Yildiz et al., 2013). Recently, a high quality assembly of the carrot genome has been reported, together with several resequenced genomes of wild and cultivated accessions of diverse origin (Iorizzo et al., 2016). This work allowed identification of the *Y* gene, which was shown to play a key regulatory role in carotenoid accumulation and shed light on the complexity of that process in the carrot storage root. Thus, *Y* can be considered as the first carrot domestication gene characterized in detail.

Previously, we reported on several Diversity Array Technology (DArT) polymorphisms showing signatures of selection upon domestication which were divided into three categories, i.e., those that were selected primarily in the cultivated carrots, representing primary domestication events, those under continuous selection from wild to eastern to western, and those differentiating western from both eastern and wild, representing secondary domestication events, likely related to traits differentiating eastern and western cultivated carrots (Grzebelus et al., 2014). One of those markers, crPt-895548, showing the most pronounced signature of selection was subsequently converted to a codominant cleaved amplified polymorphic site (CAPS) marker named *cult* (cultivated) which was shown to discriminate wild and domesticated *D. carota* gene pools (Macko-Podgórni et al., 2014). The polymorphism resulted from a 6 bp-long insertion in the cultivated carrot, comprising the *Pst*I restriction site. Here, we characterized in detail the genomic region on carrot chromosome 2 flanking the *cult* polymorphism and being under selection in the cultivated carrot gene pool. Based on the observation that the systematic difference between the wild and the cultivated carrots was limited to a very narrow region comprising a putative regulatory gene belonging to the AT-hook motif nuclear localized (AHL) family, we speculated that it was involved in the development of the carrot storage root. The selection likely operated on the standing variation, as the variant selected for in the cultivated carrot had been present in wild carrots from Central Asia, but not in wild carrots outside that region.

## Materials and Methods

### Plant Materials

To analyze nucleotide diversity (π) and pairwise population differentiation (F_ST_) we used sequencing data for a set of 29 resequenced genomes of *D. carota* (Iorizzo et al., 2016; sequence read archive accession SRP062070) comprising 11 wild, 9 eastern cultivated, and 9 western cultivated carrot accessions. For long-range PCR, we used eight plants representing wild and cultivated populations (**Table 1**) and four plants from an F_2_ (*D. carota* subsp. *commutatus* × line 2874B) preselected as homozygous ‘wild’ and ‘cultivated’ with respect to the *cult* marker. One hundred and eighty-three plants from the same F_2_ population were used to construct a genetic map and identify QTLs for root thickening. The plants were grown in the experimental field of the Institute of Plant Biology and Biotechnology at Prusy near Krakow, Poland. The seeds were sown on April 24, 2014 and roots were harvested on September 9, 2014. Head diameter and crown diameter were measured for each root immediately after harvest (**Supplementary Figure 1A**) and head to crown ratio was calculated. For RT-qPCR analysis, two orange-rooted cultivated (2874B and Kokubu Senko Oonaga) and two wild (*D. carota* subsp. *commutatus* and *D. carota* subsp. *carota*) accessions were grown in the greenhouse. The plants were harvested at three time-points, i.e., (1) 4-week-old seedlings, (2) 8- and (3) 20-week-old plants. In time point (1) whole plants were used, in time points (2) and (3), leaves, upper and lower portions of storage roots were collected separately. At each time point, samples were taken from three randomly selected plants of each accession, immediately frozen in liquid nitrogen and stored at -80°C.

**Table 1:** List of accessions used for long-range PCR amplification and sequencing of the region spanning the *cult* polymorphism.

Code	Origin	Source^a^	Country of origin	Type	*cult* variant
M1	*D. carota* subsp. *carota*	GRC11014	Greece	Wild	‘wild’
M2	*D. carota* subsp. *commutatus*	JKI-W232/07	n.a.	Wild	‘wild’
M3	2874B	IBRIB	–	Western cultivated	‘cultivated’
M4	Kokubu Senko Oonaga	Mikado kyowa Seed Co. Ltd.	Japan	Western cultivated	‘cultivated’
M5	B7262	USDA	–	Inbred	‘wild’
M6	*D. carota* subsp. *carota*	HRIGRU8716	Great Britain	Wild	‘wild’
M7	*D. carota* subsp. *carota*	HRIGRU10190	Turkey	Wild	‘wild’
M8	B9304	USDA	–	Inbred	‘cultivated’
M9	F_2_ (*D. carota* subsp. *commutatus* × 2874B)	–	–	–	‘cultivated’
M10	F_2_ (*D. carota* subsp. *commutatus* × 2874B)	–	–	–	‘cultivated’
M11	F_2_ (*D. carota* subsp. *commutatus* × 2874B)	–	–	–	‘wild’
M12	F_2_ (*D. carota* subsp. *commutatus* × 2874B)	–	–	–	‘wild’


*^a^GRC, Greek gene bank, Greece; JKI, Julius-Kuehn Institute, Germany; IBRIB, Institute of Plant Biology and Biotechnology, University of Agriculture in Krakow, Poland; USDA, United States Department of Agriculture, Madison, WI, USA; HRIGRU, Horticulture Research International – Genetic Resources Unit, UK.*

### Fluorescence *In situ* Hybridization

To obtain meiotic preparations, immature umbels of the DH1 carrot reference line (NCBI biosample accession SAMN03216637) were collected from flowering plants and fixed in Carnoy’s solution (ethanol:glacial acetic acid – 3:1). Prior to slide preparations, the umbels were washed from fixative solutions in distilled water (three times, 5 min each washing). Anthers isolated under a stereomicroscope were macerated in the enzyme mixture consisting of 4% (w/v) cellulose Onozuka R10 (Duchefa Biochemie, Haarlem, The Netherlands), 2% (w/v) pectolyase Y23 (Duchefa) and 0.04% (w/v) pectinase (Sigma), in 0.01 M citrate buffer, pH 4.8 for 40 min at 37°C. After digestion, one anther was transferred to a glass slide and preparation was performed as described by Iovene et al. (2011). Two FISH probes were used: a BAC probe DHBAC.b0022A10 specific to carrot chromosome 2 (Iorizzo et al., 2016) and a probe specific to a 37 kb-long region from 41,842,867 to 41,880,732 nt on chromosome 2 spanning the *cult* site. To prepare the *cult* probe, long PCR derived fragments (see below) covering 97% of that region were used for labeling. Both probes were labeled with either biotin-16-dUTP or digoxigenin-11-dUTP using nick translation mix (Roche Diagnostic, Mannheim, Germany) following the manufacturer’s protocol until the length of the probe fragments averaged about 100–500 bp. Labeled DNA was purified with Quick Spin G-50 Sephadex Columns (Roche Diagnostics) following the manufacturer’s protocol. FISH was carried out according to published protocols (Dong et al., 2000; Iovene et al., 2008). Carrot genomic DNA sheared up to 500 bp fragment size was used as blocking DNA in the hybridization mixture. To reduce the background signal of applied probes, 500× excess of blocking DNA was required. Biotin- and digoxigenin-labeled probes were immuno-detected with 10 μg/ml of Alexa Fluor 488-conjugated streptavidin antibody (Life Technologies) and 2 μg/ml rhodamine-conjugated anti-digoxigenin antibody (Roche Diagnostics), respectively. Chromosomes were counterstained with 1 μg/ml of 4′,6-diamidino-2-phenylindole (DAPI) in Prolong Gold antifade solution (Invitrogen, Carlsbad, CA, USA). The slides were examined with AxioImager M2 Zeiss microscope. All images were captured digitally using BV MV System (Applied Spectral Imaging) and Case Data Manager 4.0 software (ASI).

### Long-Range PCR

Amplification was carried out in 50 μl total volume containing 250 ng of genomic DNA, 15 μM of each primer, 25 mM of dNTP (Thermo Fisher Scientific), 3.5 U Expand Long Template Enzyme Mix (Roche) and 1× Expand Long Template Buffer 3 (Roche). PCR amplifications were performed in an Eppendorf Master Cycler Gradient using the following thermal conditions: 94°C (2 min), 10 cycles of 94°C (10 s), 57°C or 58°C (30 s), 68°C (6 min), 20 cycles of 94°C (15 s), 57°C or 58°C (30 s), 68°C (6 min + 20 s elongation for each successive cycle) and final elongation of 68°C (10 min). Each template was amplified using four to six primer pairs anchored in the exons of predicted genes in the investigated region (**Supplementary Table 1**). PCR products were purified using Agencourt AMPure XP purification system (Beckman Coulter) and pooled in equimolar amounts. Pooled amplicons were fragmented with NEBNext^®^ dsDNA Fragmentase^®^ (NEB) and used for library preparation (NEBNext^®^ DNA Library Prep Master Mix Set for Illumina^®^) (NEB). All samples were sequenced on one lane of MiSeq (Illumina) and assembled by a commercial service provider Genomed SA, Warsaw, Poland. Polymorphisms between cultivated and wild carrot in the coding regions were identified upon mapping to reference transcript sequences (Iorizzo et al., 2016). A codon-based test of purifying selection (Nei and Gojobori, 1986) was run using MEGA6 (Tamura et al., 2013).

### RT-qPCR

All steps of analysis followed the MIQE guidelines (Bustin et al., 2009). From each sample, total RNA was extracted using the TRIzol Plus RNA Purification Kit (Thermo Fisher Scientific) and DNA was removed with the Turbo DNA-free kit (Thermo Fisher Scientific) following the manufacturer’s protocol. Quality and quantity of RNA was determined using a NanoDrop 2000c (Thermo Scientific) and gel electrophoresis. cDNA was synthesized from 1 μg of RNA using iScript kit (Bio-Rad) and stored at -20°C. Primers for DCAR_008402 (F: CTCTATGTATCTTGTCCGCC, R: GAGATATTATGCTTGTCTGGTTC) were designed using Primer-BLAST (Ye et al., 2012) and checked with OligoAnalyzer (IDT). We used carrot actin (GenBank no. X17526.1) and ubiquitin (GenBank no. U68751.1) as reference genes, as proposed by Bowman et al. (2014). Primer efficiencies and RT-qPCR were performed as described by Bowman et al. (2014) using the StepOnePlus Real-Time PCR (Thermo Fisher Scientific). Relative expression ratios (RERs) were calculated using the ΔΔCt method (Livak and Schmittgen, 2001). ANOVA and *post hoc* Tukey HSD test were performed with R (Faraway, 2002).

### Bioinformatic Analyses

Raw reads of 29 resequenced *D. carota* accessions (Iorizzo et al., 2016) comprising 11 wild and 18 cultivated carrot accessions were cleaned by trimming low quality reads and clipping adapters using Trimmomatic 0.35 (Bolger et al., 2014) with parameters minqual = 28, minlen = 50, LEADING:28, TRAILING:28, SLIDINGWINDOW:10:28, MINLEN:50 and mapped to the reference DH1 genome (Iorizzo et al., 2016; NCBI accession LNRQ01000000) using BWA-MEM 0.7.12 (Li and Durbin, 2009). Subsequently, duplicates were marked with Samblaster 0.1.22 (Faust and Hall, 2014) and files were converted into BAM and sorted using SAMtools 1.2 (Li et al., 2009). SNPs were called using Freebayes 1.0.1 (Garrison and Marth, 2012) and filtered using vcffilter^1^ with parameters -f “QUAL > 20 and QUAL/AO > 10 and SAF > 0 and SAR > 0 and RPR > 1 and RPL > 1.” A 1 Mb sequence centered around the *cult* site (41,361,527–42,364,686) was extracted and polymorphisms were additionally filtered with parameters –max-alleles 2 –maf 0.1 –max-missing-count 0 –remove-indels, and divided into cultivated and wild populations files using VCFTools 0.1.14 (Danecek et al., 2011). Nucleotide diversity (π) and pairwise population differentiation F_ST_ were calculated using VCFTools. Linkage disequilibrium (LD) was calculated and plotted using Haploview 4.2 (Barrett et al., 2005). The vcf file was also used to identify polymorphisms (synonymous and non-synonymous SNPs present in transcribed regions) differentiating cultivated and wild carrots.

### Genotyping, Genetic Mapping, and QTL Analysis

DNA from 183 plants from the F_2_ (*D. carota* subsp. *commutatus* × line 2874B) population was extracted using a modified CTAB method (Murray and Thompson, 1980). Quality and quantity of DNA was tested using 1% agarose electrophoresis with a dilution series of λ phage DNA (10, 20, 40, 60, 80, 100, 140, and 180 ng) as a standard. Also, 200–300 ng of DNA from 20 randomly selected samples was digested with *Hin*dIII and separated using 1% agarose gel. Extracted DNA was freeze-dried and shipped to the University of Wisconsin Biotech Center, Madison (WI, USA) to perform genotyping-by-sequencing (GBS). Libraries were prepared according to Elshire et al. (2011) and run on one lane of HiSeq 2000 (Illumina) at the UW Biotech Center. The data were analyzed using TASSEL 4.3.11 essentially as described by Iorizzo et al. (2016) and filtered with plugin = GBSHapMapFiltersPlugin (mnTCov = “0.1”, mnSCov = “0.1”, mnMAF = “0.1”, mxMAF = “0.5”, mnR2 = “0.1”, mnBonP = “0.01”). The obtained vcf file was additionally filtered using VCFTools with parameters: –max-alleles 2 –min-alleles 2 –max-missing-count 10 –thin 100000 and converted into the format required for mapping using a custom Perl script. The polymorphism originally identified with the DArT marker crPt-895548 (Grzebelus et al., 2014), later referred to as *cult*, was genotyped in the codominant fashion according to the protocol developed by Macko-Podgórni et al. (2014). Mapping was performed in JoinMap 4.0 (Van Ooijen, 2006) using the regression mapping algorithm and the Haldane mapping function. SNPs showing segregation distortion (*p* < 0.001) were removed. Maps generated in round 2 were used for QTL analysis performed using Windows QTL Cartographer v.2.5 (Wang et al., 2012). The genetic map and root head diameter to crown diameter ratios of were used as input data. Composite interval mapping (CIM) with five control markers, window size of 10 cM and backward regression method was used for QTL identification. Empirical thresholds obtained from permutation tests (permutation times = 500, significance level = 0.05) were used to determine QTL significance. A putative QTL was declared significant when the LOD score was >2.5.

## Results

### Genomic Localization of the *cult* Region

**FIGURE 1:**
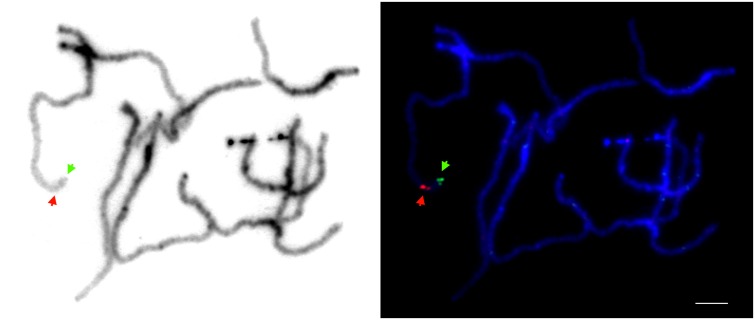
**Physical localization of the region comprising the *cult* polymorphic site on carrot chromosomes analyzed by fluorescence *in situ* hybridization.** Pachytene chromosomes were stained with DAPI (blue). Green and red signals represent the physical positions of DHBAC.b0022A10 (mapping to a distal position on the long arm of chromosome 2; Iorizzo et al., 2016) and long-range PCR amplicons spanning the *cult* region, respectively. Bar = 5 μm.

We mapped the sequence of the *cult* amplicon to the high-quality carrot genome assembly (Iorizzo et al., 2016; NCBI accession LNRQ01000000). It mapped unambiguously to the long arm of chromosome 2, position 41,862,153–41,862,461, spanning a portion of intron 1 of the gene DCAR_008402. We applied fluorescence *in situ* hybridization, with long-PCR products used as probes, as a means to confirm that it was a single copy region. The single FISH signal was present in a distal region of the long arm of chromosome 2, as expected (**Figure 1**).

### Delimitation of the Selective Sweep Range around the *cult* Polymorphic Site

**FIGURE 2:**
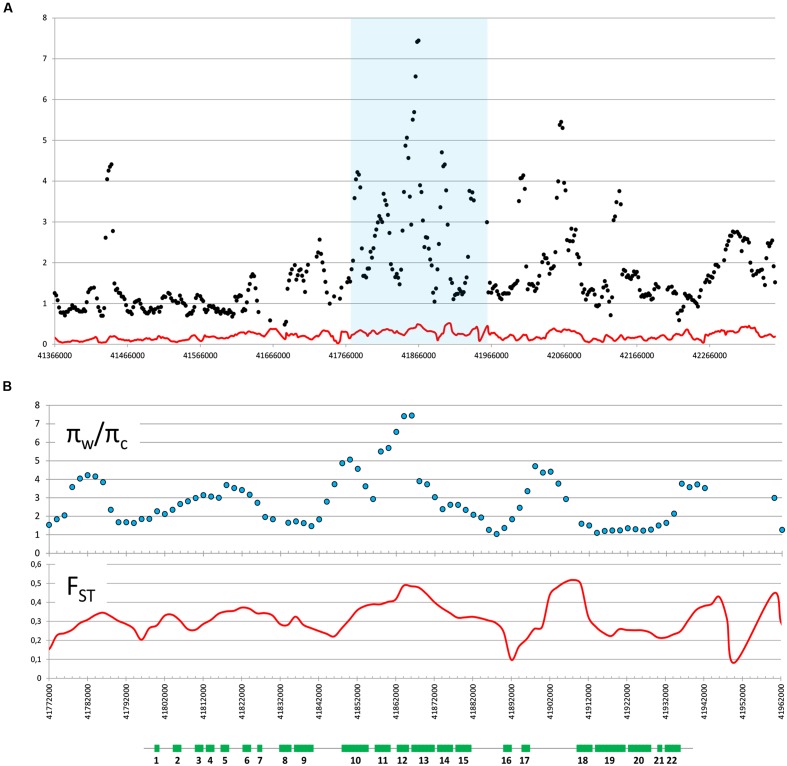
**Wild/cultivated nucleotide diversity ratio (π_w_/π_c_) and pairwise population differentiation (F_ST_).** π_w_/π_c_ (dots) and F_ST_ (red line) are graphed on carrot chromosome 2 centered around the *cult* polymorphic site in a 1 Mb **(A)** or 200 Kb **(B)** region (highlighted blue in **A**). Both parameters were calculated as average for sliding windows of size = 10 Kb and shift = 2 Kb. Schematic representation of genes (green rectangles) in the analyzed region is shown below graphs in panel **B**. Genes are numbered as in **Table 2**.

**FIGURE 3:**
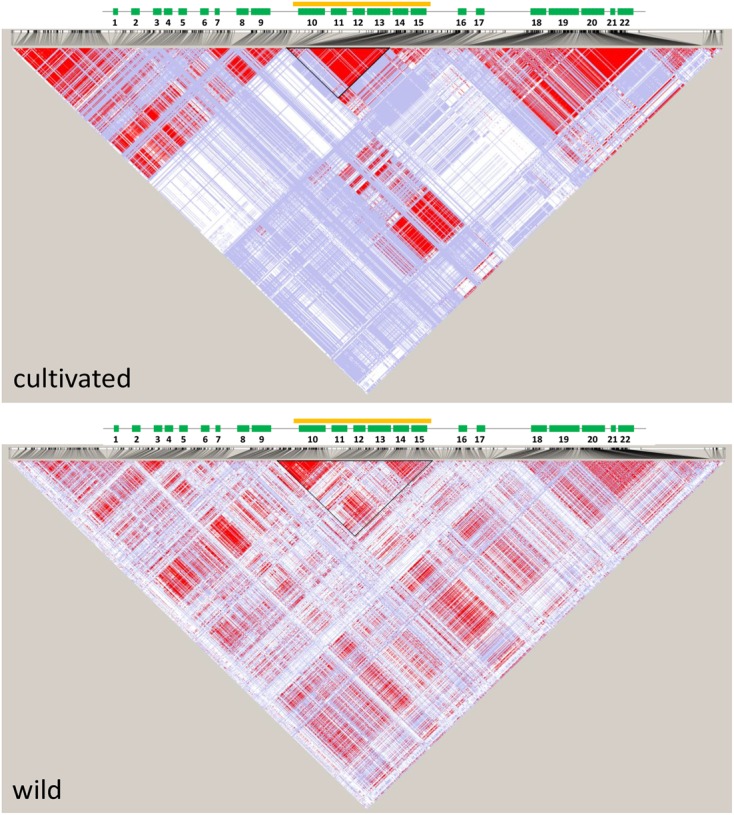
**Linkage disequilibrium (LD) plots of a 200 Kb region of carrot chromosome 2 centered around the *cult* polymorphic site for the cultivated and wild genomes.** Localization of genes is shown above (green rectangles), genes are numbered as in **Table 2**. The orange line and outlined triangle shows the region under selection.

We used previously reported genomic sequencing reads of wild and cultivated accessions of different origin (Iorizzo et al., 2016) to calculate nucleotide diversity of the wild (π_w_) and cultivated (π_c_) gene pools and the π_w_/π_c_ ratio within the 1 Mb region centered around the *cult* polymorphic site. The π_w_/π_c_ peaked around 41,858,000–41,866,000, where 7-fold decrease in nucleotide diversity was observed in the cultivated group (π_w_/π_c_ = 7.4), confirming the previously reported selective sweep around the *cult* polymorphism (Grzebelus et al., 2014). For comparison, the genomic π_w_/π_c_ ratio was 1.06, indicating a similar level of diversity in both gene pools. We also calculated pairwise population differentiation levels (F_ST_) of wild and cultivated accessions in the same region. The highest F_ST_ values were observed in regions between 41,890,000–41,900,000 (peak at 0.52) and 41,854,000 and 41,868,000 (peak at 0.48) (**Figure 2**), while the F_ST_ value estimated for the whole genome was 0.12. Furthermore, we observed elevated LD in that region in the cultivated genomes, as compared to their wild relatives (**Figure 3**). Integration of these data allowed more precise delimitation of the region of nearly 37 Kb (41,844,694–41,881,540) comprising six genes (labeled 10 to 15 on **Figure 3**), which was likely under selection upon domestication. Within this region, six genes were predicted and annotated and at least three genes encode proteins that could provide regulatory functions potentially important with respect to domestication, i.e., two protein kinases (genes DCAR_008400 and DCAR_008405) and an AHL protein (gene DCAR_008402) (**Table 2**).

**Table 2:** Gene predictions within the 150 Kb region on carrot chromosome 2 spanning the *cult* polymorphic site differentiating wild and cultivated gene pools.

No.	Gene	Coordinates	Strand	Annotation
1.	DCAR_008391	41795789–41796337	–	TCP transcription factor
2.	DCAR_008392	41800930–41801478	–	TCP transcription factor
3.	DCAR_008393	41806948–41807496	–	TCP transcription factor
4.	DCAR_008394	41809569–41811531	–	Unknown
5.	DCAR_008395	41813946–41814464	–	TCP transcription factor
6.	DCAR_008396	41820247–41820918	+	NAM (no apical meristem) protein
7.	DCAR_008397	41823800–41824521	–	Unknown
8.	DCAR_008398	41829923–41832499	+	Protein of unknown function (DUF3741)
9.	DCAR_008399	41833088–41837868	+	GMP synthase
10.^∗^	DCAR_008400	41846295–41852822	–	Serine/threonine protein kinase
11.^∗^	DCAR_008401	41855883–41859070	+	Holliday junction resolvase
12.^∗^	DCAR_008402	41861507–41864690	+	AT-hook Motif Nuclear Localized (AHL) protein carrying PPC domain (DUF296)
13.^∗^	DCAR_008403	41865356–41871238	–	Glutathione peroxidase
14.^∗^	DCAR_008404	41873168–41876690	–	Glutathione *S*-transferase
15.^∗^	DCAR_008405	41877567–41881505	–	CLAVATA1 serine/threonine protein kinase
16.	DCAR_008406	41891268–41891849	–	Translation initiation factor eIF-2B alpha subunit
17.	DCAR_008407	41895737–41897443	–	Translation initiation factor eIF-2B alpha subunit
18.	DCAR_008408	41911064–41913843	+	Translation initiation factor eIF-2B alpha subunit
19.	DCAR_008409	41916229–41922698	+	Ribosomal protein S1
20.	DCAR_008410	41924651–41930224	+	Helicase
21.	DCAR_008411	41932934–41933191	+	Unknown
22.	DCAR_008412	41934309–41937626	–	Stress responsive alpha-beta barrel protein


*The six genes annotated within the 37 Kb region under selection are marked with asterisks.*

### Structural Analysis of the Region under Selection

We used long-PCR to amplify and sequence the region spanning genes DCAR_008400 to DCAR_008405 in 12 plants representing wild and cultivated types (**Table 1**), including four F_2_ plants from the cross between the wild *D. carota* subsp. *commutatus* and the cultivated carrot line 2874B (M9 to M12 in **Table 1**). Two of the F_2_ plants were preselected as homozygous ‘wild’ and the other two as homozygous ‘cultivated’ with respect to the *cult* marker (**Supplementary Figure 1B**). As we did not observe recombination in any of the four F_2_ plants throughout the whole long-range PCR amplified region, we were able to characterize the two haplotypes corresponding to the wild and the cultivated parent and use them to search for putative functional polymorphisms. For the F_2_ plants and their parents, we observed 75 single nucleotide substitutions in the six coding regions. The two haplotypes derived from the wild and the cultivated parents were compared to SNP variants present in the remaining unrelated accessions. SNPs were present in six genes. For only one of these genes, DCAR_008402, we observed SNPs systematically differentiating the wild and the cultivated groups. Three of those substitutions were non-synonymous (**Table 3**). The inbred line B7262 carried ‘wild’ variants of these SNPs. In addition to these substitutions, the previously described insertion in intron 1 (Macko-Podgórni et al., 2014) was present in all cultivated accessions except B7262. To further investigate that relationship, we evaluated SNP variants in the three polymorphic sites of DCAR_008402, as well as the intronic indel, in the 29 resequenced genomes of *D. carota*. Again, 17 cultivated carrots both of eastern and western type carried variants attributed to the cultivated gene pool, while B7262 was confirmed to carry ‘wild’ variants. Interestingly, two Asian wild carrot accessions carried ‘cultivated’ variants in all four sites, the other three Asian wild accessions being mostly heterozygous, while no ‘cultivated’ variant was attributed to the six wild accessions of European origin (**Supplementary Table 2**).

**Table 3:** Sequence variants in the DCAR_008402 coding sequence differentiating wild and cultivated accessions.

SNP position	Nucleotide substitution		Amino acid	
		
	Wild	Cultivated	Wild	Cultivated
39	T	C	Phe	Phe
174	C	T	Gly	Gly
311^a^	A	G	Asn	Ser
522	T	G	Pro	Pro
657	T	G	Ser	Ser
858	C	T	Phe	Phe
861	C	T	Val	Val
887^a^	G	A	Ser	Asn
946	G	T	Ala	Ser
948	A	T		
976–978	–	CAG	–	Gln
996	C	T	Pro	Pro
1001^a^	T(C)^b^	G	Met(Tyr)^b^	Arg
1008	C	A	Ser	Ser


*^a^Non-synonymous substitutions systematically differentiating wild and cultivated groups.*

*^b^SNP variant identified for B7262 and *D. carota* subsp. *carota* from Turkey is shown in parentheses.*

### Structural and Functional Characteristics of a Candidate Domestication Gene

**FIGURE 4:**
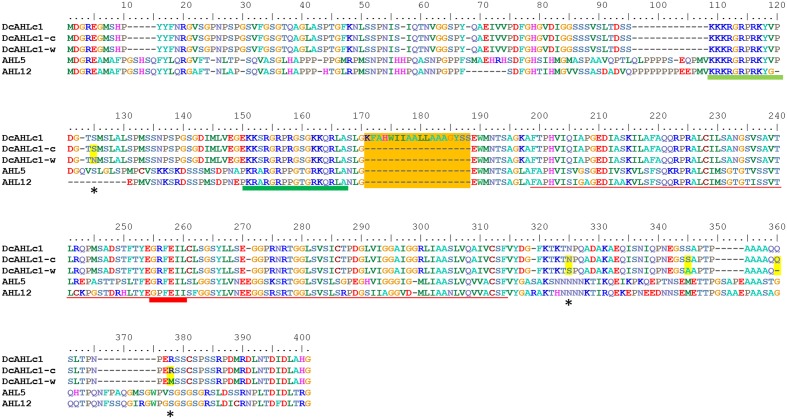
**Alignment of proteins encoded by carrot *DcAHLc1* gene and its homologs from *A. thaliana*.** Alignment of three variants showing DcAHLc1 according to the gene model, major isoforms of the cultivated and the wild type (DcAHLc1-c and DcAHLc1-w, respectively) and the two *A. thaliana* homologs, AHL5 and AHL12. Amino acid substitutions in wild and cultivated variants are indicated by stars and yellow shading, and the putative additional exon is shaded orange. The dark green and light green lines show positions of AT-hook Type-I and Type-II, respectively (Zhao et al., 2014). The red line shows the position of the PPC/DUF296 domain (Fujimoto et al., 2004) with the GRFEIL conserved motif underlined with a thicker line.

Following the results presented above, we carried out a more detailed analysis of DCAR_008402, a candidate domestication gene. It encodes a protein belonging to the AHL family of land plant specific transcription factors (Zhao et al., 2014). It can be classified into subfamily B2 of the *AHL* gene family (Zhao et al., 2014) and it clusters together with *AtAHL5* and *AtAHL12* from *Arabidopsis thaliana* (**Supplementary Figure 2**). Thus, it encodes a Type-II AHL protein containing two AT-hook motifs and Type-B PPC/DUF296 domain (**Figure 4**). Subsequently, we use the name *DcAHLc1* to denote DCAR_008402.

**FIGURE 5:**
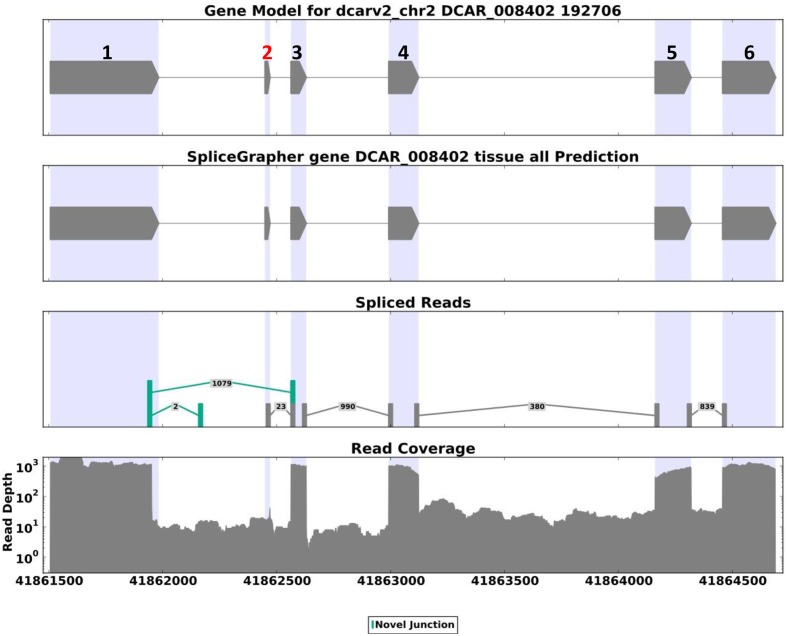
**SpliceGrapher (Rogers et al., 2012) diagram showing the structure and abundance of alternatively spliced DCAR_008402 transcripts based on the cultivated carrot transcriptome reported by Iorizzo et al. (2016).** The major isoform comprises a 3′ end-truncated exon 1 and no exon 2. There are two minor isoforms, one comprising exon 1 and a short fragment of intron 1 (supported by two reads), and the other one identical to the proposed gene model, comprising exon 2 derived from insertion in the cultivated carrot, marked red in the upper panel (supported by 23 reads).

A codon-based test for purifying selection (d_N_/d_S_) confirmed that *DcAHLc1* was likely under selection in the cultivated gene pool (**Supplementary Table 3**). In addition to the three non-synonymous single nucleotide substitutions, an insertion in intron 1 common in the cultivated carrot was observed, as reported previously (Macko-Podgórni et al., 2014). This insertion produced an additional exon, likely resulting in an alternatively spliced isoform. Indeed, transcriptome data indicated that the major *DcAHLc1* isoform did not include the additional exon, however, there was a minor isoform comprising this exon in the cultivated carrot (**Figure 5**). This exon was not present in any of the two *A. thaliana* homologs (**Figure 4**).

RNAseq data (NCBI BioProject PRJNA291977) indicated that in the cultivated carrot, *DcAHLc1* was expressed in all analyzed tissues, including fibrous and storage roots (hypocotyl, phloem, and xylem), buds and open flower, leaf and petiole, callus and germinating seeds (**Supplementary Table 4**). qRT-PCR indicated that no significant differences were observed in the total expression levels of *DcAHLc1* in seedlings, developing or mature roots and leaves of wild and cultivated *D. carota* (**Supplementary Figure 3**). This suggests that structural differences between the wild and the cultivated variant of the gene might play a role on the functionality of this gene, rather than contrasting quantitative expression patterns.

### A QTL for Root Thickening Overlaps with the *cult* Site

**FIGURE 6:**
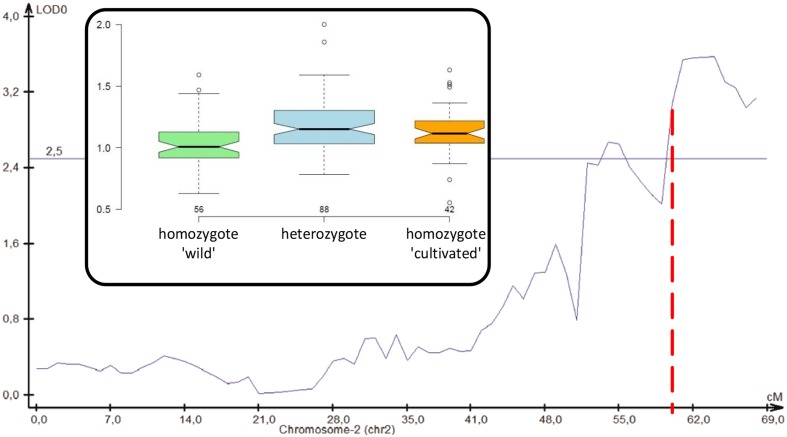
**Position of a QTL for root thickening on carrot chromosome 2.** Genetic distance is shown on the *X*-axis, LOD is on the *Y*-axis, thin horizontal line shows the significance threshold, red dashed vertical line shows the position of *DcAHLc1*. Inset: a boxplot presenting arm to crown ratio distributions in plants with respect to the *cult* marker variant.

We performed a QTL analysis for root thickening measured by the root head diameter to crown diameter ratio in a field-grown wild × cultivated F_2_ population. Five QTLs were revealed on chromosomes 2, 3, 4, 5, and 8, jointly explaining over 62% of the total variation (**Table 4**; **Supplementary Figure 4**). The QTL on chromosome 2 overlapped the *cult* site (**Figure 6**), indicating that *DcAHLc1* may possibly be involved in the development of the carrot storage root. However, QTLs on chromosomes 3, 4, and 5 had greater effect on root thickening than that on chromosome 2 (**Table 4**), likely reflecting the complexity of storage root developmental regulation.

**Table 4:** Chromosomal location and characteristics of QTLs for root thickening in *D. carota* subsp. *commutatus* × 2874B F_2_ population.

QTL ID	Chromo-some	Position (cM)	LOD value	LOD support interval	Nearest marker	% Variation explained^a^
Q1	2	64.0	3.6	60.0–68.0	2_42552923	8.53
Q2	3	44.0	6.3	33.6–53.3	3_30320926	14.44
Q3	4	34.0	6.8	14.8–34.8	4_25093202	15.50
Q4	5	39.1	7.2	28.7–48.5	5_21499391	16.33
Q5	8	40.0	3.2	43.7–44.3	8_25025402	7.62


*^a^Calculated according to the formula: (1 - 10^-^$\frac{2}{n}$^∗^LOD)^∗^100 (Broman and Sen, 2009).*

## Discussion

### Detection of the Selective Sweep on Carrot Chromosome 2

In any crop, a set of traits differentiating the cultivated form from its wild progenitor, conferring adaptation to a cultivated environment and to consumer needs, can be defined. They are collectively called the domestication syndrome (Gepts, 2014). These traits have been under continuous selection in the course of the domestication process, resulting in local decrease of variability caused by selective sweeps in genomic regions overlapping with domestication syndrome genes in cultivated populations, as compared to their wild counterparts. Very often, domestication leads to overall decrease in genetic variability, referred to as a genetic bottleneck.

In carrot, reduction of lateral root branching and biennial growth habit were pointed out as the major primary domestication syndrome traits, the latter being crucial for the development of a non-woody storage root, while further improvements comprised root quality traits, such as pigment content and flavor. Unlike most other crops, no major decrease in overall genetic diversity was reported for carrot (Iorizzo et al., 2013, 2016; Grzebelus et al., 2014), likely resulting from its predominant outcrossing mating system and the constant bidirectional gene flow between wild and cultivated populations throughout the crop breeding history until very recently. Nevertheless, it should be possible to identify local reduction in diversity around domestication syndrome genes. In our previous study, we employed a low density screen for selective sweeps using a set of 900 DArT markers and identified 27 polymorphic sites showing signatures for selection (Grzebelus et al., 2014). It employed a relatively low number of polymorphisms, hence many domestication-associated regions remained unrecognized. Given the low marker density and a likely high rate of LD in carrot as an outcrossing species, the identification of the region on chromosome 2 can be viewed as a ‘needle in a haystack’ situation. Nevertheless, the data presented here confirm the presence of a selection sweep around the original DArT polymorphism. Using multiple lines of evidence, we revealed a 37 Kb-long genomic region on chromosome 2 under selection in the cultivated carrot. There were six genes annotated in that region, but only one of them, DCAR_008402, coding for a protein belonging to the AHL family, carried polymorphisms systematically differentiating wild and cultivated gene pools.

### *DcAHLc1* as a Domestication Syndrome Candidate Gene

The *AHL* gene family is widely distributed in land plants, with individual genomes carrying several copies. Twenty-nine *AHL* paralogs were identified in *Arabidopsis thaliana* (Fujimoto et al., 2004). Plant *AHL*s were further divided into two clades; intron-less Clade A and intron-containing Clade B, comprising *AHL*s of Type I, and Types II and III, respectively (Zhao et al., 2014). It has been reported that genes belonging to the *AHL* family regulate a range of processes related to plant growth and development, including not only hypocotyl growth (Street et al., 2008; Xiao et al., 2009; Zhao et al., 2013), root development (Zhou et al., 2013), floral development (Ng et al., 2009; Gallavotti et al., 2011; Jin et al., 2011; Yun et al., 2012), but also defense against pathogens (Lu et al., 2010; Yadeta et al., 2011). It has been shown that AHL3 and AHL4 proteins act jointly as transcription factors and are involved in the regulation of vascular tissue boundaries in *A. thaliana* determined by their intercellular trafficking (Zhou et al., 2013). *AHL5* and *AHL12*, the closest homeologs to *DcAHLc1*, have not been functionally characterized in *A. thaliana*, as most other members of AHL Clade B. It seems conceivable that *DcAHLc1* is a component of a regulatory complex involved in the development of the fleshy storage root typical for the cultivated carrot. However, the mechanism can be quite complex, as possibly it requires tissue- or even cell-specific expression (Zhou et al., 2013), intercellular movement (Jang and Lee, 2014), and direct interactions between AHLs via the PPC/DUF296 domain and with other proteins (Zhao et al., 2013). Our qPCR experiments failed to reveal any significant differences in expression of *DcAHLc1*, in part because of high differences among biological replicates, therefore we speculate that modifications of the interaction or movement capability resulting from changes in the gene sequence and/or structure could be responsible for the developmental shift, although the effects of tight cell-specific expression regulation which were difficult to detect using the applied methodology cannot be excluded.

While the ‘cultivated’ variant of *DcAHLc1* was not observed in any of the European wild *D. carota*, it was present relatively frequently in the wild Asian populations. Notably, two of the wild Central Asian accessions, i.e., PI 274297 from Pakistan and PI 478369 from Xinjiang, China, were homozygous for the ‘cultivated’ variant. Convincing evidence was provided that carrot was domesticated in Central Asia (Iorizzo et al., 2013, 2016) which stands in line with reports that wild carrots from that region are capable of developing at least a primitive storage root (Vavilov, 1992). These observations imply that the mutation occurred in the wild *D. carota* from Central Asia, and selection upon domestication operated on the standing variation.

Most cultivated carrots evaluated carried the ‘cultivated’ variant of *DcAHLc1*, however, the ‘wild’ variant was present in the inbred line B7262. Previously, we observed the presence of the ‘wild’ variant mostly in primitive eastern landraces (Macko-Podgórni et al., 2014), from which the purple-rooted B7262 was derived (Simon et al., 1997). In our opinion, this observation further illustrates the complexity of the regulatory mechanism implying a possible compensatory effect, which might be provided by carrot *AHL* paralogs. Nevertheless, in advanced carrot cultivars of western type, the ‘cultivated’ variant is highly predominant. Thus, we conclude that *DcAHLc1* is a candidate domestication gene in carrot. Given the high level of gene flow postulated between the wild and the cultivated carrot throughout most of the crop history which limited the expected genetic bottleneck (Iorizzo et al., 2013), the selective sweep around *DcAHLc1* implies that it had to be under constant selection which underscores the significance of this gene for the cultivated carrot phenotype. On the basis of a QTL study, we tentatively postulate that the gene is involved in root thickening, however, the relationship has yet to be evaluated across multiple environments and populations and at a functional level. As *DcAHLc1* is expressed in most developing tissues, pleiotropic effects of its action could be expected.

## Conclusion

A DArT marker based low-density screen for selective sweeps inferred by the process of domestication across the genome of cultivated carrot revealed a highly significant polymorphism named *cult*. Here, we mapped the *cult* region to the distal portion of the long arm of chromosome 2, delimited the region under selection overlapping with the cult region to ca. 37 Kb and proposed a candidate domestication gene. The gene *DcAHLc1* belongs to the AHL family of plant regulatory proteins. The gene variant predominant in the cultivated gene pool has been selected from the wild Central Asian *D. carota*. Following the preliminary evidence from a QTL study and knowledge about the mode of function of other AHLs related to root tissue patterning, we speculate that the *DcAHLc1*-encoded protein might be involved in the transition from the woody fibrous root of wild *D. carota* to the thick fleshy root typical for cultivated carrot. It provides an interesting example of the function of a gene belonging to a relatively poorly characterized family of plant transcription factors in determination of an agronomically important trait, i.e., the carrot storage root.

## Author Contributions

AM-P and DG designed the study; GM and KS performed phenotyping; GM and KS performed genotyping; GM, MI, and DG performed mapping and QTL analyses; GM performed long-PCR, EG performed FISH; DS, MI, and PS provided resequencing data; AM-P, GM, DS, MI, and DG performed bioinformatic analyses; AM-P, EG, GM, KS, and DG drafted sections of the manuscript, MI and PS critically revised the manuscript, AM-P and DG prepared the final version of the paper. All authors read, reviewed, and approved the manuscript.

## Conflict of Interest Statement

The authors declare that the research was conducted in the absence of any commercial or financial relationships that could be construed as a potential conflict of interest.

## Footnotes

1 https://github.com/vcflib
