# Supplementary material for: Characterization of a Genomic Region under Selection in Cultivated Carrot (Daucus carota subsp. sativus) Reveals a Candidate Domestication Gene
Source: Front Plant Sci. 2017 Jan 18;8:12. doi: 10.3389/fpls.2017.00012 (PMC5241283; doi:10.3389/fpls.2017.00012)
Supplement: Supplementary file 1 [file Data_Sheet_1.PDF]

Supplementary Material

Characterization of a genomic region under selection in cultivated carrot (*Daucus carota* subsp. *sativus*) reveals a candidate domestication gene

Alicja Macko-Podgórni, Gabriela Machaj, Katarzyna Stelmach, Douglas Senalik, Ewa Grzebelus, Massimo Iorizzo, Philipp W. Simon, Dariusz Grzebelus

Corresponding Author: d.grzebelus@ogr.ur.krakow.pl

Supplementary Figures and Tables

1.1 Supplementary Figures

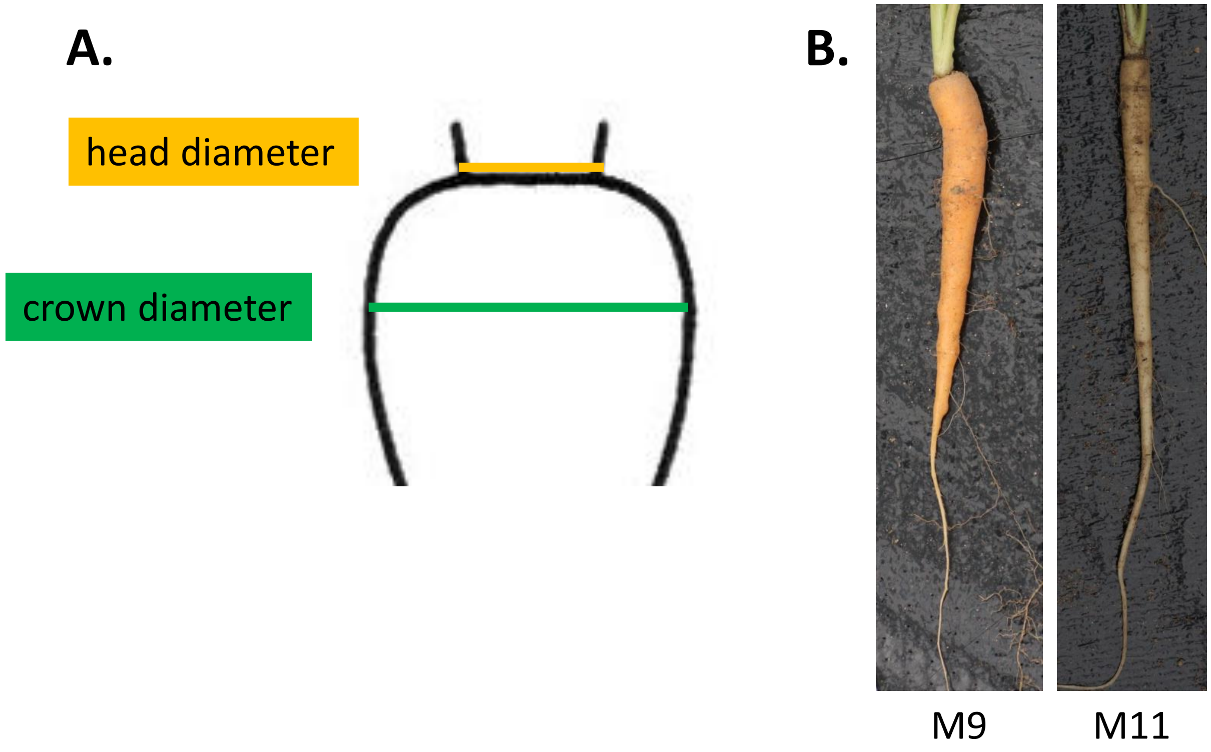

Supplementary Figure 1. Schematic representation of root measurements (A) and exemplary phenotypes of two plants from the F_2 (*D. carota* subsp. *commutatus* × 2874B) mapping population. M9 and M11 are plants genotyped as homozygous for the 'cultivated' and the 'wild' variant of the *cult* marker, which were used for long range-PCR.

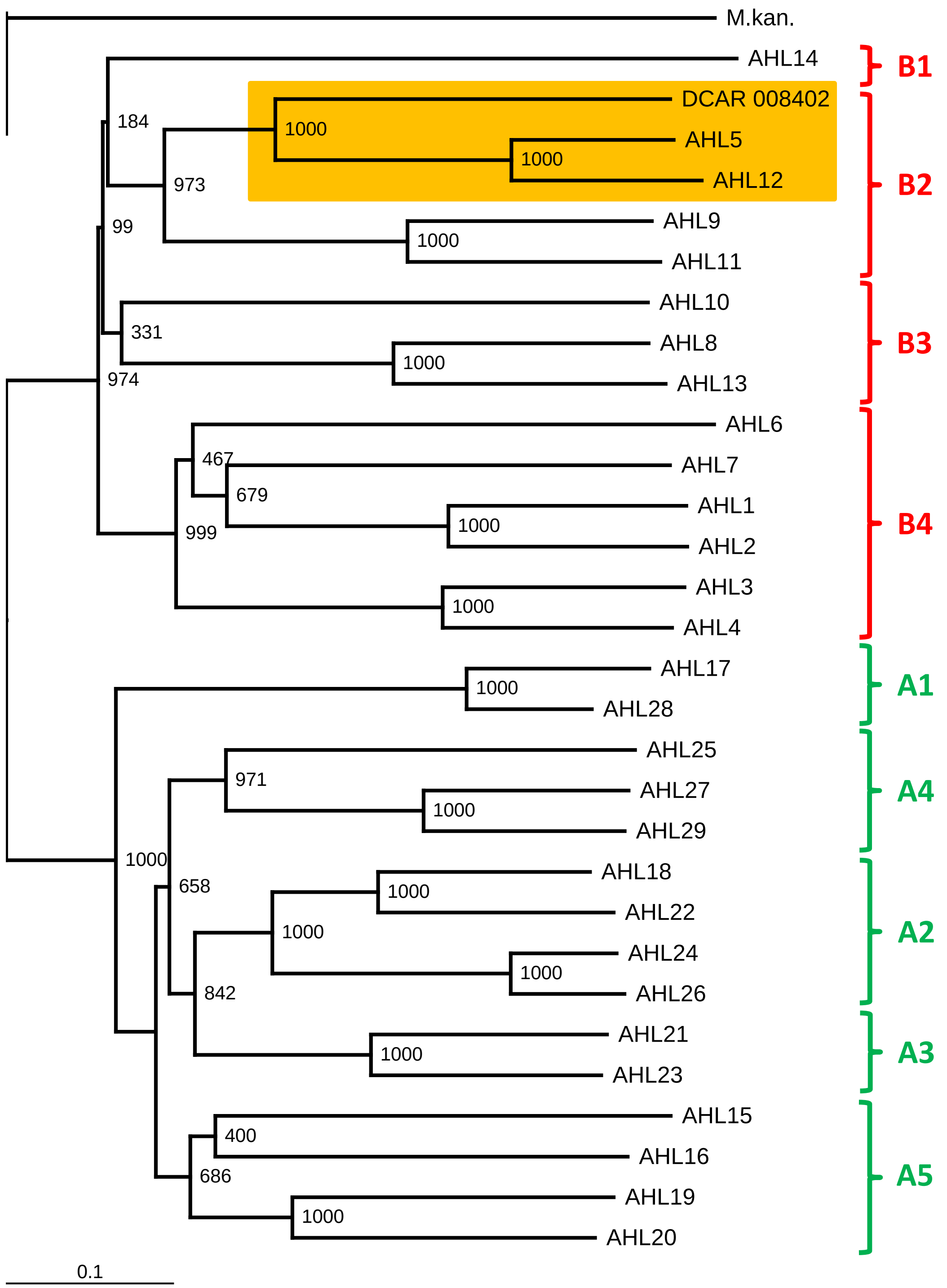

Supplementary Figure 2. Bootstrap neighbor-joining tree of the AHL family in *Arabidopsis thaliana* (after Fujimoto et al. 2004) and DCAR_008402 (DcAHLc1) from *Daucus carota*. The position of the clade comprising DCAR_008402 and two of its homeologs from *A. thaliana* (AHL5 and AHL12) is highlighted orange. Subfamilies are marked green (Clade A) and red (Clade B), classification according to Zhao et al. (2014). The AT-hook protein sequence of *Methanopyrus candleri* (AMM01827) was used to root the tree.

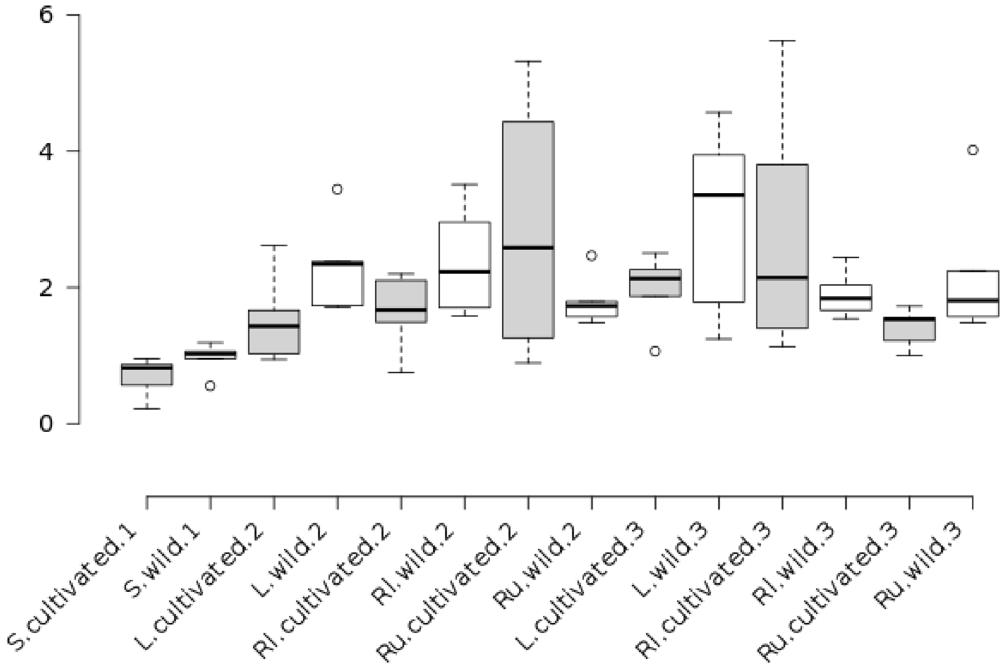

Supplementary Figure 3. Boxplot showing relative expression levels (Y axis) of DCAR_008402 (*DcAHLc1*) as measured by qPCR in five biological replicates for each tissue/plant type/time point combination. S – whole seedlings, L – leaves, Rl – roots (lower portion), Ru – roots (upper portion), 1, 2, 3 – time points (see Materials and Methods). Means are depicted by thick horizontal lines. The chart was plotted using BoxPlotR (http://boxplot.tyerslab.com/)

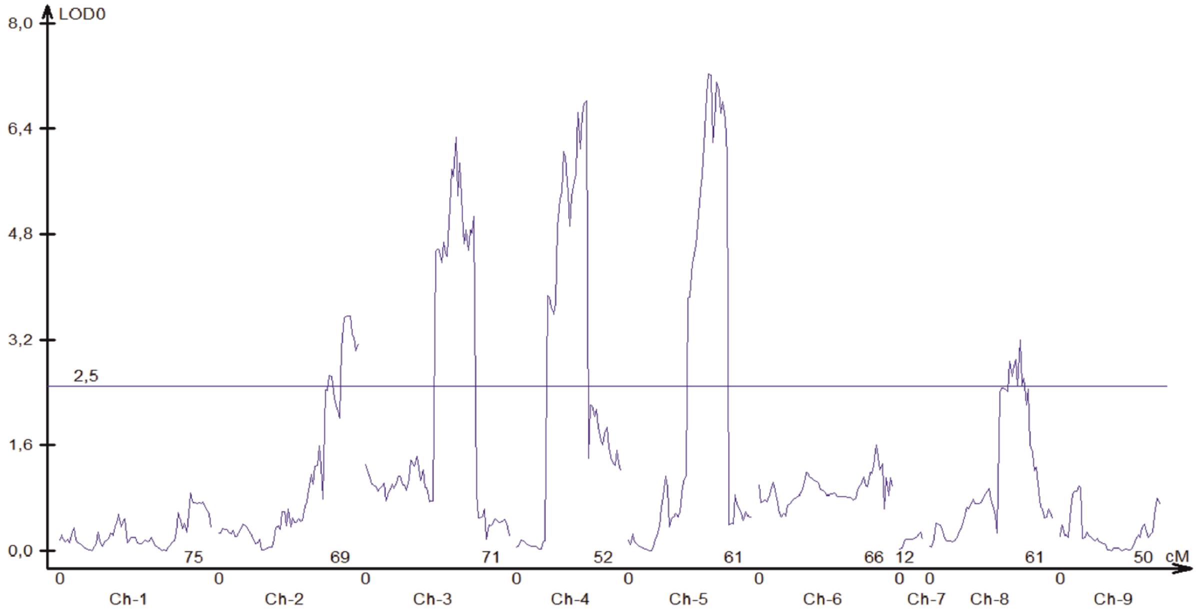

Supplementary Figure 4. Graph showing locations of QTL for root thickening along the 9 carrot chromosomes in the F_2 (*D. carota* subsp. *commutatus* × 2874B) mapping population. Genetic distance is shown on the X axis, LOD is on the Y axis, horizontal line shows the significance threshold.

1.2 Supplementary Tables

Supplementary Table 1. Primers used for long-range PCR of the carrot chromosome 2 region spanning the *cult* site

Primer pair		Primer sequence (5' -> 3')	Annealing temp. (°C)	Predicted product size (bp)	Plant Sample[a]											
					M1[b]	M2	M3	M4	M5	M6	M7	M8	M9	M10	M11	M12
A	Forward	CTTGATGTTGTCCGCAAAG	57	6194	x	x	x	x		x	x	x	x	x	x	x
	Reverse	TCTTTGGTTACCGCTTTGAT														
B	Forward	AAATCCTTTCTCAAACAAGCC	58	7592	x	x	x	x	x	x	x	x	x	x	x	x
	Reverse	AAAGGTGATGCAATCTGGTT														
C	Forward	ACTTGATGGCCACTGATTTT	58	7862	x	x	x	x	x	x	x	x	x	x	x	x
	Reverse	CTATGCTCCCACAACTTCTC														
D	Forward	TCCATATAATGGTTTCAGCCAA	57	4215	x		x	x	x	x	x	x	x	x		
	Reverse	CCTCTCCTCTCAGCATCGAG														
E	Forward	CTTCTCGTACAACTGAGCC	58	6286	x		x	x	x		x	x	x	x		
	Reverse	GGACCTAATAGTCGGTTGC														
F	Forward	CATCACCGTTGGCACTAATA	58	4569	x	x	x	x	x	x	x	x	x	x	x	x
	Reverse	TAGCGACAGAGTTGGACCAG														

[a] x indicates primer pair used in template amplification

[b] plant sample codes are as shown in Table 1.

Supplementary Table 2. Distribution of the non-synonymous SNPs and InDel variants in the gene DCAR_008402 of wild and cultivated carrots. Color codes: presence of the cultivated variant as a homozygote is highlighted yellow, presence of wild variant is highlighted green. +/- denote presence or absence of InDel, respectively. A. – Results obtained from 29 resequenced genomes of *D. carota*, B. – Results obtained from long range-PCR. Genomic coordinates of the polymorphisms are given above the columns.

A.

				**DCARv2**	**chr2 coordinates:**	41,861,817	41,862,451	41,864,499	41,864,617
**NCBI Biosample**	**Specimen Voucher**	**ID**	**Taxonomic classification**	**Common name**	**Class**	**SNP311**	**InDel cult**	**SNP887**	**SNP1001**
SAMN03766319	B7262B 349-1 USDA	I3	*D. carota* subsp. *sativus*	-	Inbred	A/A	-/-	G/G	C/C
SAMN03766317	B2566B 921-1 USDA	I1	*D. carota* subsp. *sativus*	-	Inbred	G/G	+/+	A/A	G/G
SAMN03766318	B6274B 927-1 USDA	I2	*D. carota* subsp. *sativus*	-	Inbred	G/G	+/+	A/A	G/G
SAMN03766320	B493B 920-1 USDA	I4	*D. carota* subsp. *sativus*	-	Inbred	G/G	+/+	A/A	G/G
SAMN03766321	PI 211590	C1	*D. carota* subsp. *sativus*	Land race, Afghanistan	Eastern Cultivated	G/G	+/+	A/A	G/G
SAMN03766322	PI 652188	C2	*D. carota* subsp. *sativus*	Ping Ding, China	Eastern Cultivated	G/G	+/+	A/A	G/G
SAMN03766323	PI 540422	C3	*D. carota* subsp. *sativus*	Land race, Uzbekistan	Eastern Cultivated	G/G	+/+	A/A	G/G
SAMN03766324	PI 200876	C4	*D. carota* subsp. *sativus*	Land race, Afghanistan	Eastern Cultivated	G/G	+/+	A/A	G/G
SAMN03766325	PI 652336	C5	*D. carota* subsp. *sativus*	Land race, Syria	Eastern Cultivated	G/G	+/+	A/A	G/G
SAMN03766326	PI 652374	C6	*D. carota* subsp. *sativus*	Land race, Turkey	Eastern Cultivated	G/G	+/+	A/A	G/G
SAMN03766327	PI 652136	C7	*D. carota* subsp. *sativus*	Shin Kuroda Gosun, Japan	Western cultivated	G/G	+/+	A/A	G/G
SAMN03766328	Embrapa LOTE 39/06	C8	*D. carota* subsp. *sativus*	Brasilia, Brazil	Western cultivated	G/G	+/+	A/A	G/G
SAMN03766329	PI 261648	C9	*D. carota* subsp. *sativus*	Kokubu, Netherlands	Western cultivated	G/G	+/+	A/A	G/G
SAMN03766330	PI 643114	C10	*D. carota* subsp. *sativus*	White Belgian, USA	Western cultivated	G/G	+/+	A/A	G/G
SAMN03766331	PI 451755	C11	*D. carota* subsp. *sativus*	Lange gele stomper, Netherlands	Western cultivated	G/G	+/+	A/A	G/G
SAMN03766332	PI 264232	C12	*D. carota* subsp. *sativus*	Chantenay Red Cored, France	Western cultivated	G/G	+/+	A/A	G/G
SAMN03766333	PI 632391	C13	*D. carota* subsp. *sativus*	Long Imperator 58, USA	Western cultivated	G/G	+/+	A/A	G/G
SAMN03766334	PI 187235	C14	*D. carota* subsp. *sativus*	Nantes no. 1, Belgium	Western cultivated	G/G	+/+	A/A	G/G
SAMN03766338	PI 478369	W4	*D. carota* subsp. *carota*	China	Eastern wild	G/G	+/+	A/A	G/G
SAMN03766339	PI 274297	W8	*D. carota* subsp. *carota*	Pakistan	Eastern wild	G/G	+/+	A/A	G/G
SAMN03766337	PI 652358	W7	*D. carota* subsp. *carota*	Turkey	Eastern wild	G/G	-/-	A/A	T/T
SAMN03766335	Ames 27395	W5	*D. carota* subsp. *carota*	Uzbekistan	Eastern wild	G/A	+/-	A/G	G/G
SAMN03766343	PI 652393	W6	*D. carota* subsp. *carota*	Turkey	Eastern wild	G/A	+/-	A/G	G/T
SAMN03766342	PI 502244	W1	*D. carota* subsp. *carota*	Portugal	Western wild	A/A	-/-	C/C	T/T
SAMN03766350	Ames 26408	W2	*D. carota* subsp. *carota*	Portugal	Western wild	A/A	-/-	G/G	T/C
SAMN03766336	PI 478861	W3	*D. carota* subsp. *carota*	France	Western wild	A/A	-/-	G/G	T/T
SAMN03766344	Ames 26381	Ssp1	*D. carota* subsp. *gummifer*	Portugal	Western wild	A/A	-/-	G/C	T/T
SAMN03766351	Ames 31194	Ssp3	*D. carota* subsp. *gummifer*	France	Western wild	A/A	-/-	G/C	T/C
SAMN03766341	PI 478883	Ssp4	*D. carota* subsp. *gummifer*	France	Western wild	A/A	-/-	G/G	T/T

B.

Source	Specimen Voucher	ID	Taxonomic classification	Common name	Class	SNP311	InDel cult	SNP887	SNP1001
Long range-PCR	B7262B-USDA	M5	*D. carota* subsp. *sativus*		inbred	A/A	-/-	G/G	C/C
Long range-PCR	B9304B-USDA	M8	*D. carota* subsp. *sativus*		inbred	G/G	+/+	A/A	G/G
Long range-PCR	2874B-IBRIB	M3	*D. carota* subsp. *sativus*		Western cultivated	G/G	+/+	A/A	G/G
Long range-PCR		M4	*D. carota* subsp. *sativus*	Kokubu Senko Oonaga	Western cultivated	G/G	+/+	A/A	G/G
Long range-PCR		M9	F2 (*D. carota* subsp. *commutatus* × 2874B)	-	wild/cultivated hybrid	G/G	+/+	A/A	G/G
Long range-PCR		M10	F2 (*D. carota* subsp. *commutatus* × 2874B)	-	wild/cultivated hybrid	G/G	+/+	A/A	G/G
Long range-PCR		M11	F2 (*D. carota* subsp. *commutatus* × 2874B)	-	wild/cultivated hybrid	A/A	-/-	G/G	T/T
Long range-PCR		M12	F2 (*D. carota* subsp. *commutatus* × 2874B)	-	wild/cultivated hybrid	A/A	-/-	G/G	T/T
Long range-PCR		M1	*D. carota* subsp. *carota*	Greece	Eastern wild	A/A	-/-	G/G	T/T
Long range-PCR		M2	*D. carota* subsp. *commutatus*	n.a.	Western wild	A/A	-/-	G/G	T/T
Long range-PCR		M6	*D. carota* subsp. *carota*	Great Britain	Western wild	A/A	-/-	G/G	T/T
Long range-PCR		M7	*D. carota* subsp. *carota*	Turkey	Eastern wild	G/A	+/-	A/G	G/C

Supplementary Table 3. Codon-based Test of Purifying Selection for analysis between DCAR_008402 sequences of eight plants (M1-M8; see Table 1) and the DH1 reference line (Iorizzo et al. 2016). The probability of rejecting the null hypothesis of strict-neutrality (dN = dS) in favor of the alternative hypothesis (dN < dS) (below diagonal) is shown. Values of P less than 0.05 are considered significant at the 5% level and are highlighted yellow. The test statistic (dS - dN) is shown above the diagonal. dS and dN are the numbers of synonymous and nonsynonymous substitutions per site, respectively. The variance of the difference was computed using the analytical method. Analyses were conducted using the Nei-Gojobori method. The analysis involved nine nucleotide sequences. All ambiguous positions were removed for each sequence pair. There were a total of 363 positions in the final dataset. Evolutionary analyses were conducted in MEGA6.

	M3	M8	M4	M7	M1	M2	M6	M5	DH
M3		0	0	2.803	0.908	2.538	1.832	2.803	0
M8	1		0	2.803	0.908	2.538	1.832	2.803	0
M4	1	1		2.803	0.908	2.538	1.832	2.803	0
M7	0.003	0.003	0.003		2.33	2.975	2.329	0	2.803
M1	0.183	0.183	0.183	0.011		2.597	1.862	2.33	0.908
M2	0.006	0.006	0.006	0.002	0.005		0.9	2.975	2.538
M6	0.035	0.035	0.035	0.011	0.033	0.185		2.329	1.832
M5	0.003	0.003	0.003	1	0.011	0.002	0.011		2.803
DH	1	1	1	0.003	0.183	0.006	0.035	0.003	

Supplementary Table 4. Tissue-specific expression pattern of *DcAHLc1* in cultivated carrot DH1, as revealed by transcriptome sequencing (Iorizzo et al. 2016; NCBI BioProject PRJNA291977).

Tissue	FPKM
0.5 mm bud (Bud)	62.0548
Bracts from flower, not opened (Br1)	13.0507
Whole flower, not opened (Fl1)	2.24165
Bracts from flower at anthesis (Br2)	1.90804
Whole flower at anthesis (Fl2)	11.4339
0.5-1 cm young leaf (Lf1)	17.3461
2-2.5 cm leaf (Lf2)	5.32363
7-8 cm leaf (Lf3)	3.91196
10 cm petiole (Pet)	5.79121
Hypocotyle from 4 cm root, 1.5 cm diameter (Hyp)	21.7862
Phloem from 4 cm root, 1.5 cm diameter (Phl)	22.2475
Xylem from 4 cm root, 1.5 cm diameter (Xyl)	23.3053
Fiber roots (Rtf)	17.1082
Callus (Cal)	18.0365
2-2.5 cm leaf at reversible wilting point (Ls1)	4.95971
Petiole from 2-2.5 cm leaf at reversible wilting point (Pts)	5.11808
7-8 cm leaf at reversible wilting point (Ls2)	1.92885
Whole storage root (Rts)	2.87678
Etiolated leaf (Lfe)	13.3379
Seed at the beginning of germination (Sd1)	8.73659
